# Global identification of bursicon-regulated genes in *Drosophila melanogaster*

**DOI:** 10.1186/1471-2164-9-424

**Published:** 2008-09-18

**Authors:** Shiheng An, Songjie Wang, Lawrence I Gilbert, Brenda Beerntsen, Mark Ellersieck, Qisheng Song

**Affiliations:** 1 Division of Plant Sciences, University of Missouri, Columbia, MO 65211, USA; 2 Department of Biology, University of North Carolina, Chapel Hill, NC 27599-3280, USA; 3 Department of Veterinary Pathobiology, University of Missouri, Columbia, MO 65211, USA; 4 Experiment Station Statistics, University of Missouri, Columbia, MO 65211, USA

## Abstract

**Background:**

Bursicon is a heterodimer neuropeptide responsible for regulating cuticle sclerotization and wing expansion in several insect species. Recent studies indicate that the action of bursicon is mediated by a specific G protein-coupled receptor DLGR2 and the cAMP/PKA signaling pathway. However, little is known regarding the genes that are regulated by bursicon. The identification of bursicon-regulated genes is the focus of this investigation.

**Results:**

We used DNA microarray analysis to identify bursicon-regulated genes in neck-ligated flies (*Drosophila melanogaster) *that received recombinant bursicon (r-bursicon). Fifty four genes were found to be regulated by bursicon 1 h post r-bursicon injection, 52 being up-regulated and 2 down-regulated while 33 genes were influenced by r-bursicon 3 h post-injection (24 up-regulated and 9 down-regulated genes). Analysis of these genes by inference from the fly database  revealed that these genes encode proteins with diverse functions, including cell signaling, gene transcription, DNA/RNA binding, ion trafficking, proteolysis-peptidolysis, metabolism, cytoskeleton formation, immune response and cell-adhesion. Twenty eight genes randomly selected from the microarray-identified list were verified by real time PCR (qPCR) which supported the microarray data. Temporal response studies of 13 identified and verified genes by qPCR revealed that the temporal expression patterns of these genes are consistent with the microarray data.

**Conclusion:**

Using r-bursicon, we identified 87 genes that are regulated by bursicon, 30 of which have no previously known function. Most importantly, all genes randomly selected from the microarray-identified list were verified by real time PCR. Temporal analysis of 13 verified genes revealed that the expression of these genes was indeed induced by bursicon and correlated well with the cuticle sclerotization process. The composite data suggest that these genes play important roles in regulating the cuticle sclerotization and wing expansion processes. The data obtained here will form the basis for future studies aimed at elucidating the exact mechanisms upstream from the secretion of bursicon and its binding to target cells.

## Background

Molting is a process common to all arthropods, during which a larger new cuticle is synthesized and the old one is digested and cast off (ecdysis) allowing the animal to grow. Studies on ecdysial behavior in insects showed that at least six different hormones are released in an orderly manner during the molting cycle to regulate the synthesis and sclerotization (hardening and tanning) of new cuticle [1-5]. The final hormone released in this cascade, the neuropeptide bursicon, was found to trigger sclerotization of the new cuticle four decades ago using the then novel neck-ligated blowfly bioassay in which the ligated flies are injected with an extract of the fused thoracic/abdominal ganglion or with hemolymph collected shortly after adult emergence [5-7]. Bursicon activity in the thoracic-abdominal ganglion was strikingly higher than that of the brain [5]. Using this bioassay, bursicon activity was identified in diverse insect orders including Diptera, Orthoptera, Hemiptera, Coleoptera and Lepidoptera [5]. Bursicon also stimulated wing expansion in newly emerged lepidopteran adults [8-10].

Previously, bursicon was thought to be a monomeric neuropeptide with a molecular size ranging from 30 to 60 kDa [5]. More recently, functional bursicon was shown to be a heterodimer consisting of two cystine knot subunits, referred to as bursicon α (CG13419) and bursicon β (CG15284)[11,12]. In *Drosophila melanogaster*, bursicon acts via a specific G protein-coupled receptor (GPCR) DLGR2, encoded by the *rickets *gene [9]. DLGR2, once activated, is hypothesized to activate the cAMP/PKA signaling pathway [13]. Recombinant bursicon (r-bursicon) heterodimer was also found to bind to DLGR2 with high affinity, to stimulate cAMP production *in vitro *and to then initiate cuticle sclerotization in the ligated fruit fly bioassay *in vivo *[11,12]. Mutation of the bursicon α gene and receptor *ricket*s gene caused defects in cuticle sclerotization and wing expansion [8,9]. Of great interest is the work of Davis et al (2007) who showed that tyrosine hydroxylase, the enzyme mediating the conversion of tyrosine to DOPA in the metabolic pathway leading to cuticle tanning, is activated by PKA via bursicon stimulation of DLGR2 [14]. A gene silencing study revealed that injection of the double-stranded bursicon α RNA into *Bombyx mori *(silkworm) pupae significantly reduced the level of bursicon α mRNA, resulting in decreased wing expansion in the newly emerged adult moth [10]. However, little is known about the signaling pathway downstream of the bursicon receptor DLGR2 and adenylate cyclase, as well as the genes regulated by bursicon. Here, we report the functional analysis and identification of the genes affected by the injection of r-bursicon into the thorax of ligated *D. melanogaster *just after ecdysis to the adult.

## Results

### Functional analysis of r-bursicon in neck-ligated flies

**Figure 1:**
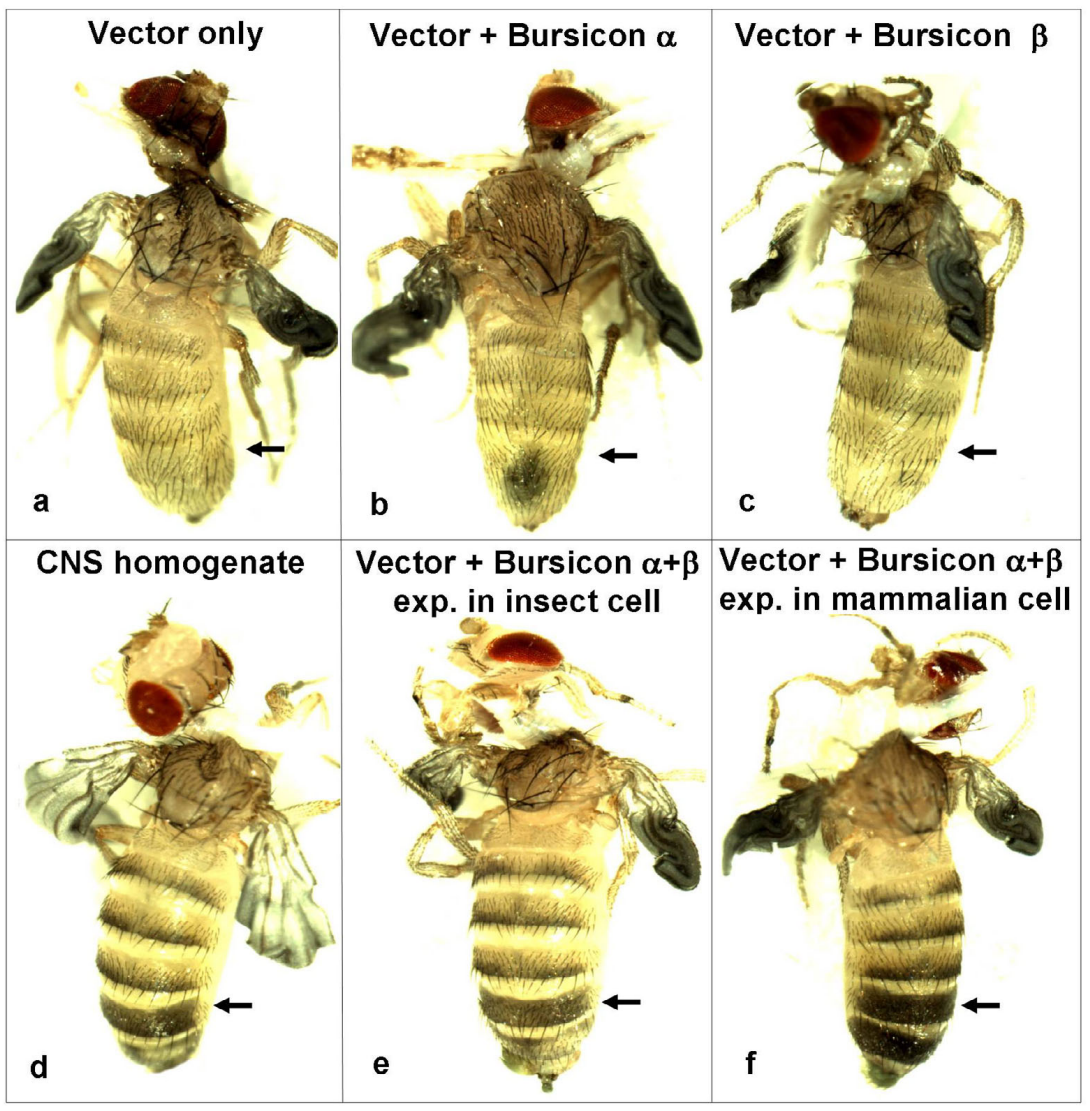
**Functional assay of the r-bursicon heterodimer in ligated flies**. Newly emerged flies were ligated between the head and thorax at emergence. These flies with unsclerotized cuticle at 1 h after neck-ligation were injected with 0.5 μl of cell culture transfected with blank pcDNA 3.1 vector as a sham control (a) or with the purified r-bursicon α (b) or r-bursicon β (c) or r-bursicon heterodimer expressed in insect High Five™ cells (e) and in mammalian HEK293 cells (f). A CNS homogenate (0.5 CNS equivalent/fly) from newly emerged flies was used as a positive control (d). The arrow indicates the area with the unsclerotized cuticle (light color) in control (a-c) and the sclerotized cuticle (darkened) in the animals injected with r-bursicon heterodimer.

**Figure 2:**
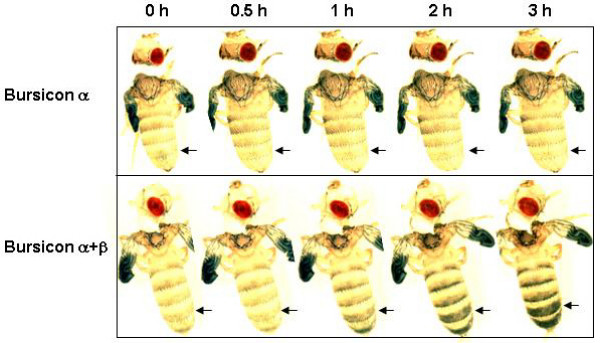
**Temporal response of the ligated flies to the r-bursicon heterodimer**. Newly emerged flies were ligated between the head and thorax at emergence. These flies with unsclerotized cuticle at 1 h after neck-ligation were injected with 0.5 μl of the purified r-bursicon α (negative control) or r-bursicon heterodimer expressed in mammalian HEK293 cells. The cuticle sclerotization process was photographed at the indicated time periods after injection. The arrow indicates the area with the unsclerotized cuticle (light color) in control and the sclerotized cuticle (darkened) in the animals injected with r-bursicon heterodimer.

The r-bursicon protein, expressed in mammalian HEK293 cells or in insect High Five™ cells, was assayed for bursicon activity using the neck-ligated fly assay. Three hours after injection (Fig. 1), no sign of cuticle sclerotization was observed in abdomens injected with 0.5 μl of the supernatant from a cell culture transfected with blank vector (Fig. 1a, sham control) or with r-bursicon α (Fig. 1b, negative control) or with r-bursicon β alone (Fig. 1c, negative control). Cuticle tanning was detected only in those abdomens injected with the homogenate of the CNS (the fused thoracic/abdominal ganglion without brain) (0.5 CNS equivalent/fly) (Fig. 1d, positive control) or the purified r-bursicon heterodimer expressed in insect High Five™ cells (Fig. 1e) and mammalian HEK293 cells (Fig. 1f). When the neck-ligated flies were injected with r-bursicon and photographed at the indicated time periods (Fig. 2), cuticle sclerotization was not visible at 30 min, slightly visible at 1 h and almost complete by 3 h post r-bursicon injection, data consistent with past observations [11,12]. Additionally, these results suggest that the r-bursicon heterodimer expressed in both cell cultures has strong cuticle sclerotization activity in the neck-ligated fly assay and could therefore be used to identify early and late bursicon-regulated genes in the subsequent microarray analyses.

### DNA microarray identification of early and late bursicon-regulated genes

To identify bursicon-regulated genes, gene expression experiments were performed at 1 and 3 h after bursicon administration. We first determined the transcriptional profile of the 18,952 transcripts (the transcript numbers in the gene chip) in the ligated flies injected with r-bursicon α (negative control) at the two time points noted above i.e. 1 h (early response) and 3 h (late response) post r-bursicon injection. The results showed that 12,057 transcripts were present in the control 1 h post r-bursicon injection, which corresponds to 63.61% of the total number of described *Drosophila *genes while 12,048 transcripts (63.57% of total genes) were present in the control 3 h post r-bursicon injection. We then compared gene-expression profiles between the control and the r-bursicon heterodimer-injected flies at these two time points. Statistical filtering of the microarray data revealed that the expression of 54 transcripts was up- or down-regulated by the r-bursicon heterodimer by at least 2 fold (*P *< 0.05) at 1 h and 33 transcripts at 3 h post r-bursicon injection (Table 1). More upregulated genes were present at 1 h (52 genes) than at 3 h (24 genes) while more down-regulated genes were revealed at 3 h (9 genes) than at 1 h (2 genes) after r-bursicon injection. Surprisingly, only two genes, CG30080-RA and CG32491 (mdg4) involved in gene transcription, were up-regulated at both 1 and 3 h after r-bursicon injection.

**Table 1:** Microarray identification of the genes that are up- and down-regulated by r-bursicon in the neck-ligated fly assay at 1 and 3 h post r-bursicon injection (≥ 2.0 folds, p < 0.05).

		1 h			3 h	
Function	Transcript	Gene name or function domain	Fold	Transcript	Gene name or function domain	Fold
Signaling	CG2849-RB	Ras-related protein	3.9	CG17226-RA	Odorant receptor 59c	-2.7
	CG6407-RA	Wnt oncogene analog 5	2.3			
	CG11348-RB	Nicotinic acetylchol. receptor	2.1			
	CG32659-RA	Tenascin accessory (EGF- Domain)	2.1			
	CG2346-RA	FMRFamide-related	2.0			
	CG10125-RA	Zero population growth (Innexin domain)	2.0			
	CG6386-RA	Ballchen (Kinase domain)	2.0			
Immune response	CG10146-RA	Attacin	7.9	CG31193-RA	Humoral factor Turandot X	3.8
	CG33202-RB	(Immunoglobulin domain)	2.3	CG31691-RA	Humoral factor Turandot F	2.3
	CG18372-RA	Attacin	2.0	CG31508-RA	Humoral factor Turandot C	2.2
				CG1878-RA	Cecropin B	2.3
Channel & transporter	CG9500-RA	(Fibrinogen-related domains)	2.4	HDC14466	Magnesium/cobalt transporter	3.0
	CG12348-RA	Shaker (BTB/POZ domain)	2.1	CG10369	Inward rectifier K^+ ^channel	2.3
	CG4110-RA	Pickpocket 11 (Sodium channel)	2.0	CG33098-RC	Ca^2+ ^sensors & Ca^2+ ^signal MODs	2.1
	CG1522-RA	Cacophony (Ion transport protein)	2.0	HDC02744	Iron-sulfur cluster-binding protein	2.0
	CG14872-RB	(Lipocalin/cytosolic fatty-acid binding protein)	2.1	CG10844	Ryanodine-sensitive Ca^2+^-release channel	-2.6
	CG8221-RA	Amyrel (Alpha amylase)	-2.5			
Transcription	**CG30080-** **RA**	Iduronate-2- like sulfatase	**3.0**	**CG30080-** **RA**	Iduronate-2- like sulfatase	**2.1**
	**CG32491-** **RA**	Modifier of mdg4	**2.4**	**CG32491-** **RA**	Modifier of mdg4	**2.4**
	CG1624-RC	Dappled (Zinc binding domain)	2.0	CG11501-RA	Transcriptional regulator	9.6
	CG6157-RA	Discontinuous actin hexagon (Zinc binding domain)	2.0			
	CG8260-RA	(BTB/POZ domain)	2.0			
DNA/RNA binding	CG3497-RA	Suppressor of Hairless (DNA binding domain)	2.4	CG12924-RA	Eukary. Sm & Sm-like (LSm) protein	3.2
	CG3019-RA	Suppressor of white-apricot	2.1	CG32438-RA	SMC5 proteins (ATPase family)	-2.2
	CG8920-RA	Tudor domain	2.1			
	CG7776-RA	Enhancer of Polycomb	2.0			
	CG10897-RA	Toutatis (Methyl-CpG binding domains)	2.0			
	CG10327-RC	TBPH (RNA recognition motif)	2.0			
	CG3238-RA	(DUF889 domain)	2.6			
Metabolic enzyme	CG12660-RA	Retinal degeneration A (Phorbol-esters/diacylglycerol binding domain)	2.5			
	CG13927-RA	γ- glutamyl carboxylase	2.3			
	CG10140-RA	Chitin binding Peritrophin-A domain	2.2			
	CG6822-RB	Ergic53(Legume lectin domain)	2.1			
	CG4181-RA	Glutathione S transferase	2.1			
	CG3649-RA	Sugar transporter domain	2.0			
Cytoskeletal compnt	CG13338-RA	Cuticular protein 50Ca (Chitin-binding domain)	2.1	CG32050-RA	Titin	2.0
Cell adhesion	CG3938-RE	Cyclin E	2.2	CG16826-RA	Lipoprotein	2.2
				CG7294-RA	Trophinin	2.0
Proteolysis& peptidolysis				CG4793-RB	Trypsin-like serine protease	4.9
				CG11066	Serine-type endopeptidase	2.9
				CG30287-RA	Trypsin-like serine protease	2.1
Unknown function	CG30482-RA	N	2.8	CG7985-RA	N	2.6
	CT32157	N	2.4	HDC18092	N	2.6
	CG16863-RA	N	2.3	HDC15090	N	2.4
	HDC03087	N	2.2	HDC13939	N	2.3
	HDC16822	N	2.2	HDC15545	N	2.2
	CG32027-RA	N	2.2	HDC05639	N	2.0
	HDC09490	N	2.2	HDC14431	N	-2.0
	LD33458	N	2.2	HDC09972	N	-2.1
	CG2217-RA	N	2.1	CT33997	N	-2.2
	CG17816-RA	N	2.1	HDC18592	N	-2.5
	CG40172-RA	N	2.1	CG4440-RA	N	-2.5
	CG31813-RA	N	2.1	S.CX001941	N	-4.0
	GH12319	N	2.0			
	CG32132-RA	N	2.0			
	CT29521	N	2.0			
	CG15699-RA	N	2.0			
	CG40386-RA	N	2.1			
	HDC10707	N	-2.2			

The genes in bold were detected at both 1 and 3 h post r-bursicon injection. The name in parenthesis indicates a functional domain.

N: gene with no known function associated with it.

**Figure 3:**
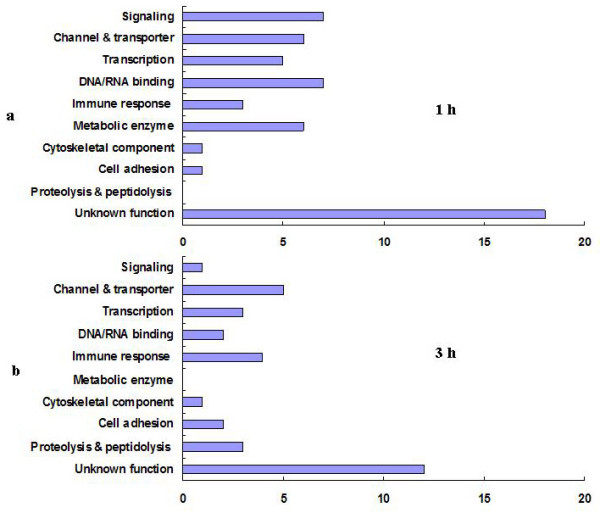
**Functional classification of the bursicon-regulated genes at 1 and 3 h post r-bursicon injection**. The classification is based on gene ontology data in the FlyBase in conjunction with a homolog search using the BLAST data in FLIGHT and functional protein domains in InterPro. The numbers along the horizontal axis indicate the transcript numbers.

Annotation of 54 genes identified 1 h post r-bursicon injection using FlyBase revealed that proteins encoded by these genes belong to diverse functional categories. Among the proteins with known function, the largest group contains those genes involved with DNA/RNA binding proteins and signaling, each with 7 proteins. The second largest groups contain those genes involved with channel/transporter and metabolic enzymes with 6 proteins each. In addition, 5 transcription factors, 3 immune response factors, one cytoskeletal component and one cell-adhesion gene product were also identified. On the other hand, 18 out of 54 bursicon-regulated genes (33.3%) encode proteins with as yet unknown functions (Fig. 3a).

Microarray analysis revealed that only 33 transcripts were regulated by bursicon at 3 h post r-bursicon injection. Among these transcripts, the channel/transporter is the largest group with 5 proteins. The second largest group is the immune response factors with 4 proteins. Transcription factors and proteolysis/peptidolysis transcripts, each encoding 3 proteins, present the third largest groups followed by 2 DNA/RNA binding proteins, 2 cell adhesion proteins, one cytoskelethal component and a signaling protein. Twelve out of 33 bursicon regulated genes (36%) encode proteins with unknown functions (Fig. 3b). Before discussing the possible function of some of these genes in the action of bursicon e.g. cuticle sclerotization and wing expansion, it was important to verify the results of the microarray analyses and for this we chose real-time (q)PCR analysis.

### Validation by qPCR

**Figure 4:**
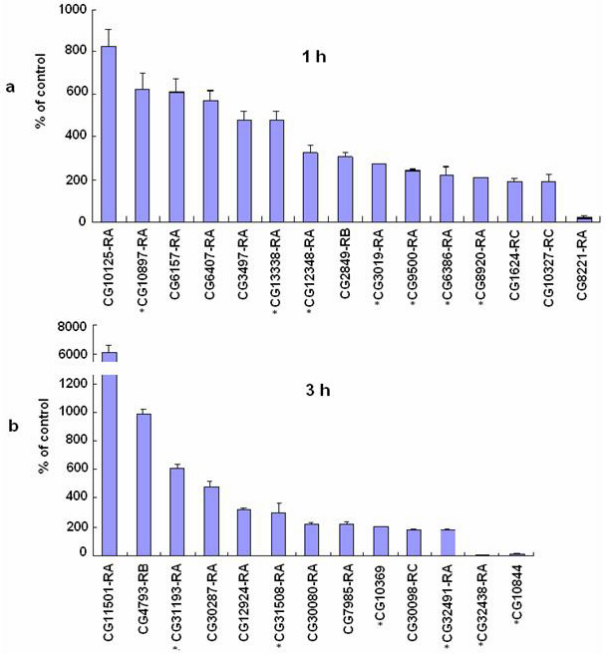
**qPCR verification of the randomly selected bursicon-regulated genes identified by microarray**. The ligated flies were injected with 0.5 μl r-bursicon heterodimer and the control flies received r-bursicon α protein only. See Methods for RNA extraction, PCR amplification, reverse transcription and mRNA quantification protocols. a. Genes regulated 1 h after r-bursicon injection. b. Genes regulated 3 h after r-bursicon injection. The data represent the mean ± SE of three biological samples. *: these genes were randomly selected for temporal transcriptional profile analysis in Fig. 5 and Fig. 6.

Specific primers (Table 2) were designed to 28 randomly selected bursicon-regulated genes (15 from 1 h, and 13 from 3 h, post r-bursicon injection)  and were utilized for corroborating estimates of gene transcription using qPCR. The RNA samples used were identical to those used for the microarray, i.e. extracted from the neck-ligated flies either 1 or 3 h post r-bursicon injection. The qPCR results revealed that all selected genes were verifiable (Fig. 4), i.e., up- or down-regulated by r-bursicon by at least 2 fold in the qPCR analysis, a result similar to that obtained for the same genes using microarray analysis (Table 1).

**Figure 5:**
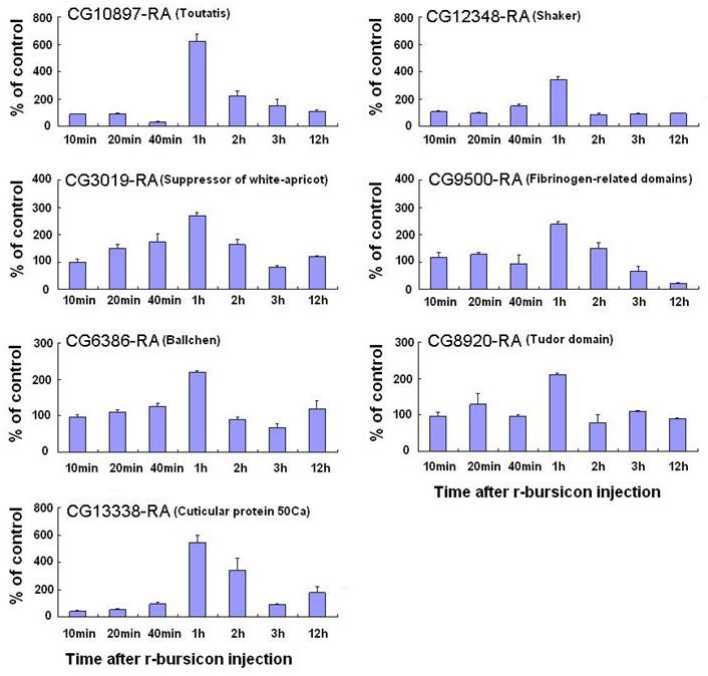
**Temporal transcriptional profiles of bursicon-regulated genes selected from 1 h microarray data**. The neck-ligated flies were injected with 0.5 μl r-bursicon heterodimer and the control flies received r-bursicon α protein only. Total RNA was extracted from the treated and control flies at the indicated time periods post r-bursicon injection and subjected to qPCR analysis as described in Methods. The data represent the mean ± SE of three biological samples.

**Figure 6:**
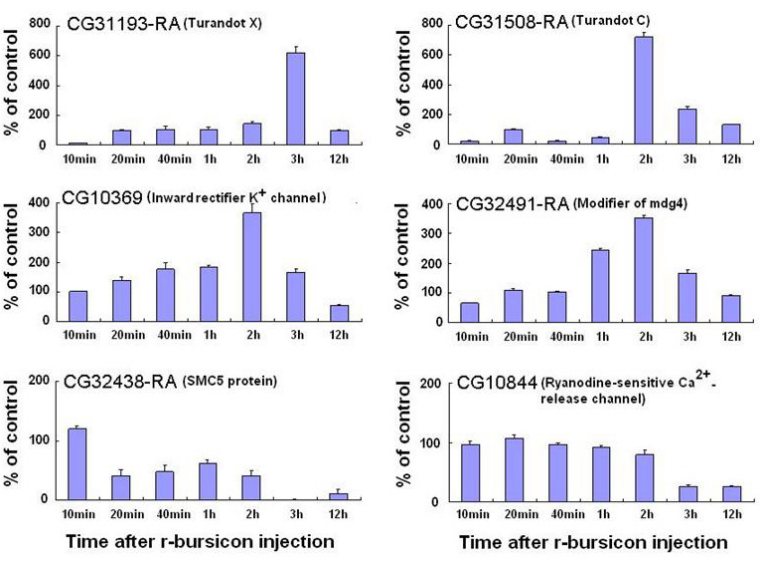
**Temporal transcriptional profiles of bursicon-regulated genes selected from 3 h microarray data**. The neck-ligated flies were injected with 0.5 μl r-bursicon heterodimer and the control flies received r-bursicon α protein only. Total RNA was extracted from the treated and control flies at the indicated time periods post r-bursicon injection and subjected to qPCR analysis as described in Methods. The data represent the mean ± SE of three biological samples.

**Table 2:** Gene specific primers for qPCR.

Gene name	Forward sequence	Reverse sequence
Genes selected from 1 h post r-bursicon injection		
CG10125-RA	5'-CTACCGTAATGCCTTGCTGTC-3'	5'-AGCAGGCAAAGGTAGTCGTATAT-3'
CG10897-RA	5'-TCCCTCATCCTTCAACGAG-3'	5'-CAATCCCTTGGGTATCTGCT-3'
CG6157-RA	5'-GCCCATCAGTGCTTTCTGCT-3'	5'-TAGCATCTGTCGCTGAGGCGTG-3'
CG6407-RA	5'-TTTGGCAACCAGGTGGAGCAG-3'	5'-GCCTTGACGAAGCCAGTCT-3'
CG3497-RA	5'-TATCCACCGACAAGGCTGAGTA-3'	5'-GGCGTAAAGTTGTCGCCACTCAG-3'
CG12348-RA	5'-CACTTTGAACCCATTCCTCAC-3'	5'-TCATTTCTAAGCGGGTCAAAGTA-3'
CG2849-RB	5'-TGGGAAAGTCCGCCCTCAC-3'	5'-TACTTCCTCGCCGTCCAGCA-3'
CG3019-RA	5'-ACCTTCACCAGCAAACCCGTGCT-3'	5'-TCGCTGCGTTGACGAATGCT-3'
CG9500-RA	5'-GACTTTGAAGGACAGACACG-3'	5'-AGATGCCGAATAGATTACTG-3'
CG6386-RA	5'-CTGTTTCCGAAGGGAGTGC-3'	5'-TGCTTTCCTGGCTGTTGCTG-3'
CG8920-RA	5'-ATACAAGCACCGTCTATCA-3'	5'-CGGCGTTACTTTCAATCTG-3'
CG1624-RC	5'-CAGCAATCCGAAGATGTCG-3'	5'-ACAGCGGACTGTTGCGATGCT-3'
CG10327-RC	5'-GTTCGCTCCAACGAGGGCAGACT-3'	5'-GGCAAGCCGAGCACGATGAG-3'
CG8221-RA	5'-ATCCCTACGGCATCAGTCG-3'	5'-ATCTGACGCCAACGATGCT-3'
Genes selected from 3 h post r-bursicon injection		
CG11501-RA	5'-ACTATCCCAACGGCTGCGAAGTG-3'	5'-GGCAGCCTGGTGACTTTGAT-3'
CG4793-RB	5'-ATCGCCTTTCCATTCTGCT-3'	5'-GTAGCGGTTGATTGTCAGC-3'
CG31193-RA	5'-TGGCAGCTTGCTAATATGCG-3'	5'-ATAAAAAGCGATTAGCTGCGG-3'
CG30287-RA	5'-TGCGTTTGGGTGATTACGA-3'	5'-TTTATGCGGGATTCAGTGC-3'
CG12924-RA	5'-CCATTGGAACTCCTAACGAAGTG-3'	5'-GTGATACCCAACTCCTTGAG-3'
CG31508-RA	5'-TTTGCTTGGGCTATTCTGAC-3'	5'-CAGTATGCTCCTGAATGGATCT-3'
CG30080-RA	5'-ATGGAGTGCAGAGCTGTTCC-3'	5'-GCCGTGATGAAGAAGCCAAT-3'
CG7985-RA	5'-CGATACGACAGCCTCAGCA-3'	5'-GACAGCGTTATCCTTGAGC-3'
CG10369	5'-CGAGGGCTGTATGGTGAGC-3'	5'-GTGCGTTTGTCCACGATGAT-3'
CG33098-RC	5'-ATGTCGATGGATCCATCGG-3'	5'-TCTCCTCGGGCAGCGTATA-3'
CG32491-RA	5'-CCCACGAAATCTGAACCTGAC-3'	5'-CGCTCGTATTTGCCGCAGTCT-3'
CG32438-RA	5'-GCAAAGATTTCGTATCCTACA-3'	5'-GGTAGACAAGCCATTAGAG-3'
CG10844	5'-GGTGGCAATGGAGTAGGCGAT-3'	5'-GCAACGCCAATAACATCGC-3'

To gain further insight into how the activity of the bursicon-regulated genes may change between 1 and 3 h post-injection, qPCR analysis was carried out on 13 genes selected from the list of 28 verifiable genes. The transcriptional profiles of these genes change over time. As expected, all 7 genes selected from the flies 1 h after r-bursicon injection were up-regulated maximally at 1 h, declined sharply thereafter and returned to the basal level by 3 h (Fig. 5). These genes include CG10897-RA, CG12348-RA, CG3019-RA, CG9500-RA, CG6386-RA, CG8920-RA, and CG13338-RA. Among 6 randomly selected genes from flies 3 h after r-bursicon injection, four were up-regulated maximally at 2 or 3 h, but not at 1 h, while 2 down-regulated genes reached their lowest levels after 1 h or later (Fig. 6). These 4 up-regulated genes include CG31193-RA, CG31508-RA, CG10369, and CG32491-RA while the 2 down-regulated genes were CG32438-RA and CG10844. The temporal response patterns of these 13 genes obtained by qPCR analysis (Fig. 5, Fig. 6) were consistent with the microarray data (Table 1).

## Discussion

The composite data indicate that the genes described above as being regulated by bursicon are associated with cuticle sclerotization and wing expansion in *Drosophila*. Among the total 87 transcripts identified at 1 and 3 h post r-bursicon injection, 57 showed significant similarities to known proteins or functional domains in the *Drosophila *data base while 30 did not. In *Drosophila*, bursicon binds with high affinity and specificity to the DLGR2 that is encoded by *rickets *[9]. DLGR2, once activated leads to the activation of the cAMP/PKA signaling pathway [11-13]. However, the signaling pathway downstream of PKA remains conjectural. Among the 87 bursicon-regulated genes we identified, 8 genes were found to control cell signaling (Fig. 3). This group includes a Ras-related protein (CG2849-RB), a G-protein activator-odorant receptor 59C (CG17226-RA) etc (Table 1). Ras proteins are considered to be very important molecular switches for a wide variety of signaling pathways that control such processes as cytoskeletal integrity, proliferation, cell adhesion, apoptosis, and cell migration [15]. Ras-related protein is often activated via phosphorylation by PKA. In yeast the Ras signaling pathway controls cell growth via PKA, resulting in the regulation of the elongation step of the RNA polymerase II transcription process [15]. Perhaps in *Drosophila *the Ras-related protein CG2849-RB identified in the microarray analysis (Table 1) might be activated directly by PKA i.e. act as a downstream component of PKA in the bursicon signaling pathway.

Bursicon is a neuropeptide that not only induces cuticle hardening and tanning but also regulates wing expansion and subsequent epidermal cell death [13]. Ras proteins have the ability to activate transforming signals as well as signals that regulate apoptosis [16]. In the Rat1 fibroblast cell line for example, the activated H-Ras^R12 ^is a potent inducer of apoptosis in response to serum starvation [17] and that may also be the case in the insect wing. In addition, microarray analysis also detected a FMRFamide (CG2346-RA), which in the clam is thought to play an important role in the regulation of heart rate, muscle contraction and blood pressure [18]. Such controls are necessary for wing expansion and FMRFamide may play an analogous role in the insect. Our microarray analyses also detected three antibacterial peptides, attacin-like (CG10146-RA and CG18372-RA) and cecropin (CG1878-RA). Although no direct association between bursicon and antibacterial peptides is obvious, the newly ecdysed insect is soft (before cuticle tanning and hardening) and more susceptible to injury. Perhaps a more vigorous antibacterial defense is necessary at this time when there may be perforations in the soft cuticle due to predators or simple contact injuries.

Three humoral factor *turandot *genes (*turandot *C-CG31508-RA, *turandot *X-CG31193-RA, *turandot *F-CG31691-RA) are also induced by bursicon (Table 1). The *turandot *gene family is considered to be immune-related in insects, and is induced under a wide range of adverse conditions such as infection, heat, oxidizing agents, wounding and aging [19]. However, under normal conditions, some *turandot *genes, such as *turandot *C, *turandot *F, etc, are expressed during specific developmental periods, indicating that these genes are associated with normal physiological morphogenetic processes [20]. Our results showed that three *turandot *genes (C, X and F) can be induced in the ligated flies injected with r-bursicon, suggesting that these genes are not only involved in specific response reactions to abnormal conditions, but may also be involved in the cuticle sclerotization process.

Using crude thoracic/abdominal ganglion homogenates, Mills and Whitehead demonstrated that bursicon enhanced the uptake of tyrosine by hemocytes [21]. This result is consistent with the result of Post [22]. They speculated that one of bursicon's functions was to increase the membrane permeability of hemocytes to tyrosine which would then be converted to N-acetyldopamine. Although we do not have direct evidence to support this claim, the microarray data did reveal that 11 channels/transporters were regulated by bursicon. These channel proteins/transporters may be involved in the uptake of tyrosine by hemocytes, the earliest precursor for tanning agent biosynthesis.

Cuticle sclerotization is a complex process, during which many genes detected in our studies are induced. The induction of these genes requires corresponding transcription-associated factors and DNA/RNA binding proteins. The present results identified several transcription-associated genes and DNA/RNA binding proteins (Table 1). For example, CG12924 encodes eukaryotic Sm and Sm-like (LSm) proteins involving pre-mRNA splicing, telomere replication, and mRNA degradation [23]. Most interesting, a trans-splicing *Drosophila *gene *mod (mdg4) *(CG32491) was found to be up-regulated by bursicon in our microarray screening. The *mod(mdg4) *gene of *D. melanogaster *has been reported to encode a chromatin protein and has been independently identified through mutations isolated for their effects on position effect variegation (PEV), the properties of insulator sequences, correct path finding of growing nerve cells, meiotic pairing of chromosomes, and apoptosis [24]. Besides the above mentioned genes, there are 11 other transcription-related genes (Table 1) detected in our microarray analysis. These genes possibly play pivotal roles in regulating the expression of genes encoding enzymes required for the cuticle sclerotization process.

Sclerotization is a metabolic process, during which enzymes mediate a significant number of reactions. These enzymes include diphenoloxidases, laccases, peroxidase, tyrosine hydroxylase (TH), dopa decarboxylase (DDC) etc [3]. It is a little surprising that our microarray analysis did not detect transcriptional changes of these enzymes. However, it should be pointed out that that not all genes are regulated at the transcriptional level. For example, TH mediates the conversion of tyrosine to dihydroxyphenylalanine (dopa) and is a key enzyme during the tanning process. However, TH mRNA level is at its highest 24 h before eclosion and remains unchanged until 12 hours after eclosion [14]. It seems that the activity of TH during the sclerotization process is not regulated at the transcriptional level, TH being transiently activated during cuticle sclerotization by a post-translational mechanism, i.e. phosphorylation by PKA [14]. Post-translational mechanisms may be in force here. Another example is DDC, which catalyzes the conversion of dopa to dopamine, a compound of central importance for sclerotization. The DDC mRNA level peaks at 24 hours before eclosion and decreases thereafter. It is almost unchanged or decreases minutely from 0 h to 3 h after eclosion. DDC is transcribed and translated before eclosion to ensure that enzyme activity is present when substrate becomes available [14]. Therefore, it is not surprising that microarray analysis does not detect the changes in some genes encoding enzymes whose activation is required for the normal processes of tanning and sclerotization. Most interestly, our microrray analysis identified 6 metabolic enzymes whose transcripts were up-regulated significantly at 1 h, but not at 3 h after r-bursicon administration, suggesting that these enzymes may be actively involved in the early sclerotization process, perhaps in the synthesis of agents required for tanning and hardening.

Sclerotization is a process of cross-linking protein to protein chains, chitin to chitin chains, and protein to chitin chains [3]. Our microarray analysis also detected a cuticular protein gene 50Ca (CG13338-RA) with a chitin-binding domain and a gene, CG10140-RA that appears to be involved in chitin metabolism. Although their exact role in cuticle formation is not known, the possibility exists that they function to bind chitin and/or play important roles in chitin metabolism. Tanning requires energy in the form of ATP (endergonic) and the CG3649-RA gene detected in the microarray analysis has a sugar transporter domain and may help supply metabolic energy for melanization. It is therefore evident that further research is necessary to identify with certainty those genes involved with specific steps in the tanning and sclerotization processes. Our data are tantalizing but only suggestive at this point.

Early studies indicate that the brain triggers the release of bursicon from the fused thoracic/abdominal ganglion to the hemolymph by nervous stimuli leading to cuticle sclerotization [5]. More recent investigations revealed that both bursicon protein and transcripts are present in the thoracic/abdominal ganglion, but not in the brain, of *D. melanogaster *[11,12] and *Musca domestica *[25], thus confirming the early observation [5]. Cuticle sclerotization is a brief but complex process occurring after each molt and the genes involved in this process are not expected to be present in large number. The 87 genes identified in the microarray analysis reflect this estimation. Although the functions and interrelationship of these genes in the cuticle sclerotization process is conjectural at present, the microarray data provide a foundation for further investigation of the functions of these identified genes, and such studies may allow a better understanding of the mechanisms of the cuticle sclerotization process in insects and other arthropods. Sclerotization of the exoskeleton is a primary reason for the success of insects on this planet and the series of reactions constituting sclerotization and its control affects more animal species than any other series of phylum-specific reactions. It is most worthy of future research and we believe that the present study lays the basis for such investigations at another level.

## Conclusion

Bursicon is a neuropeptide that regulates cuticle sclerotization and wing expansion in insects, critical processes for insect survival. In these processes, many genes are up- and down-regulated. In this report, we demonstrated the presence of a set of genes that are sensitive to r-bursicon stimulation and the data were verified by qPCR analysis. This gene set supplies unique and intriguing candidates for a more complete understanding of bursicon action by the use of specific gene deletion and over-expression experiments that may lead to a functional dissection of some of these genes in the cuticle sclerotization process.

## Methods

### Experimental insects

*D. melanogaster *(wild type^*ore*^) were reared on artificial blue diet (Fisher Scientific) at 24°C under constant darkness.

### Expression of recombinant bursicon (r-bursicon)

The central nervous system (CNS) (the fused thoracic/abdominal ganglion) was dissected under Ringers' solution (3.6 mM NaCl, 54.3 mM KCl, 8.0 mM CaCl_2_, and 28.3 mM MgCl_2_) from pharate adults which are believed to have high levels of bursicon α and β transcripts [11,12]. Total RNA was extracted from the CNS using Trizol reagent (Invitrogen) according to the manufacturer's instructions. To obtain the full length *Drosophila *bursicon α and β sequences by PCR, the open reading frames of bursicon α and β were amplified using a gene specific forward primer with a *Xho*I restriction site and a reverse primer with a *BamH*1 restriction site (Bursicon α: forward primer 5'-CTCGAGATGCTGCGCCACCTGCTCCG-3'; reverse primer 5'-GGATCCTTGCAGAGCAATGCGCCGGA-3'. Bursicon β: forward primer 5'-CTCGAGATGCATGTCCAGGAACTGCT-3'; reverse primer 5'-GGATCCACGTGTGAAATCGCCACATT-3'), cloned into the PGEM-T-Easy vector (Promega), and sequenced again for confirmation of correct insertion.

To express the *Drosophila *r-bursicon in HEK293 cells, bursicon α and β cDNAs were isolated from the PGEM-T-Easy vector using *Xho*I and *BamH*1 and ligated into the pcDNA3.1 expression vector predigested with *Xho*I and *BamH*1. The pcDNA3.1 vector containing bursicon α or β cDNA was further sequenced for confirmation of correct insertion.

The pcDNA3.1 plasmid (2 μg) containing bursicon α or β was used to transfect mammalian HEK293 cells either individually or simultaneously using the SatisFection™ Transfection Reagent (Stratagene). After 16 h initial transfection, the serum-free DMEM cell culture medium was replaced with the same medium containing 10% fetal bovine serum and the transfected cells were incubated for additional 24 h. The cell medium was then replaced with serum-free DMEM medium and cultured for 48 h. At the end of incubation, the medium was collected and centrifuged at 2000 × g to remove cell debris [11,12].

r-Bursicon α and β proteins were also expressed in insect High Five™ cells using the Bac-to-Bac baculovirus expression system according to the manufacturer's protocol (catalog #10359, Invitrogen). In brief, bursicon α and β cDNAs were each ligated into pFastBac™ donor plasmids. The purified pFastBac™ constructs were used to transform DH10Bac™ *E. coli *cells to generate recombinant bacmid. The recombinant bacmid DNA was analyzed using PCR for confirmation of recombination and used to transfect insect High Five™ cells to produce recombinant baculovirus. The recombinant baculoviruses were used to infect insect High Five™ cells to express the r-bursicon protein. The expressed recombinant protein in medium was collected by centrifugation at 2000 × g.

The expressed r-bursicon proteins in mammalian HEK293 cells and in insect High Five™ cells were purified using Ni-NTA His-bind -resin (QIAGEN) and verified by Western blot using a His-tag antibody before the bursicon bioassay.

The r-bursicon was assessed for its hormonal activity in the ligated fly assay using adults ligated between the head and thorax with dental floss immediately after ecdysis. After 1 h, 0.5 μl (120 ng/μl) of the recombinant protein containing bursicon α or bursicon β (control) or the bursicon α and β heterodimer was injected into the thorax-abdomens of ligated flies with untanned cuticle using a glass needle mounted on a microinjection system. The medium which had been passed over the Ni-NTA His bind resin (QIAGEN) from mammalian HEK293 cells transfected with blank pcDNA3.1 plasmid or from insect High Five™ cells transfected with blank bacmid was used as a sham control. The CNS extract of newly emerged flies was used as a positive control (0.5 CNS equivalent/fly). Abdominal cuticle sclerotization was evaluated at the indicated time points (0, 0.5, 1, 2 and 3 h) after injection and photographed using a Leica MZ16 microscope with a Q-imaging digital camera and MicroPublisher 5.0 software.

### DNA microarray chip

The GeneChip^® ^Drosophila Genome 2.0 Array (Affymetrix) covering over 500,000 datum points to measure the expression of 18,952 transcripts and variants was used in this study. The design of the new array was based largely on the content from the recent Annotation (release 3.1) of the *D. melanogaster *genome by Flybase, the Berkeley *Drosophila *Genome Project (BDGP) and other published gene predictions from the *Drosophila *Research community.

### Target preparation and microarray analysis

The neck-ligated flies were injected with r-bursicon α and β heterodimer (experimental) or with r-bursicon α or r-bursicon β subunit only (control) as described above. The whole fly was transferred into 1.0 ml of Trizol (Invitrogen) after 1 h and 3 h treatment with bursicon and stored at -80°C. RNA extraction was performed according to the manufacturer's instructions. Quantity and quality of total RNA were determined by RNA agarose gel electrophoresis and optical density measurement at 260 and 280 nm.

One microgram of total RNA was used to make the biotin-labeled antisense RNA (aRNA) using the MessageAmp II-Biotin Enhanced Single Round aRNA amplification kit (Ambion) following the manufacturer's procedures. Briefly, the total RNA was reverse transcribed to first strand cDNA with an oligo(dT) primer bearing a 5'-T7 promoter using ArrayScript reverse transcriptase. The first strand cDNA then underwent second-strand synthesis and purification to become the template for in vitro transcription. The biotin-labeled aRNA was synthesized using T7 RNA transcriptase with biotin-NTP mix. After purification, the aRNA was fragmented in 1× fragmentation buffer at 94°C for 35 min. Ten micrograms of fragmented aRNA in 200 μl of hybridization solution was hybridized to the *Drosophila *Genome 2.0 genechip (Affymetrix) at 45°C for 20 h. After hybridization, the chips were washed and stained with R-phycoerythrin-streptavidin on an Affymetrix fluidics station 450 using fluidics protocol Midi_euk2 v3. The image data were acquired by an Affymetrix Genechip scanner 3000 using the GCOS 1.4 software. The raw data were normalized and analyzed by using AMADA software [26]. The experiments were repeated three times with different batches of total RNA extracted from bursicon-injected and control flies. Three biological replicates were used to perform an unpaired t test [t test for independent samples with separate variance estimates (two-sided P)]. The *P *value of the unpaired t test (*P*) therefore reflects the probability at which the null hypothesis (no difference in the expression of a given gene between experimental and control samples) is rejected.

### qPCR analysis of bursicon-regulated genes

Total RNA was extracted from the treated and control flies using the Trizol reagent (Invitrogen) according to the manufacturer's instructions. First-strand cDNA was synthesized from 2 μg DNAse-treated total RNA using an oligo-dT_20 _as primer and superscript™III reverse transcriptase as enzyme (Invitrogen).

To verify the bursicon-regulated genes identified by DNA microarray analysis, we randomly selected 28 genes (14 up-regulated and 1 down-regulated genes from 1 h and 11 up-regulated and 2 down-regulated genes from 3 h post r-bursicon injection) for estimation of gene transcription using qPCR and designed gene specific primers (Table 2). The RNA samples used were identical to those used in the microarray, i.e. extracted from the ligated flies at 1 h and 3 h after r-bursicon injection.

To investigate the temporal response of the microarray identified and qPCR verified genes, 13 genes selected from the list of 28 verifiable genes were analyzed by qPCR at the indicated time periods (10, 20, 40 min, 1, 2, 3, 12 h) post r-bursicon injection.

PCR amplification and analysis were carried out on an Applied Biosystems (ABI) 7500 Fast Real-Time PCR System. The final reaction volume was 25 μl using ABI SYBR green Supermix (ABI). The PCR program was: hold at 95°C for 10 min and then at 95°C for 15 seconds and 60°C for 1 min, repeating 40 times. The specificity of the SYBR green PCR signal was further confirmed by a melting curve analysis and agarose gel electrophoresis. The mRNA expression was quantified using the comparative CT (Cross Threshold, the PCR cycle number that crosses the signal threshold) method [27]. The CT of the housekeeping gene rp49 was subtracted from the CT of the target gene to obtain ΔCT. The normalized fold changes of the target gene mRNA expression were expressed as 2^-ΔΔCT^, where ΔΔCT is equal to ΔCT_treated sample _-ΔCT_control_.

## Authors' contributions

SA carried out the Microarray and Real-time PCR analysis, drafted and helped revise the manuscript. SW generated the microarray sample and analyzed the transcript data. BB was responsible for analysis of the real-time PCR data. ME was responsible for analysis of the microarray data. LIG and QS designed the study, revised the manuscript and assessed the results.
